# Supplementary material for: Photochemical Generation of Methyl Chloride from Humic Aicd: Impacts of Precursor Concentration, Solution pH, Solution Salinity and Ferric Ion
Source: Int J Environ Res Public Health. 2020 Jan 13;17(2):503. doi: 10.3390/ijerph17020503 (PMC7013589; doi:10.3390/ijerph17020503)
Supplement: Supplementary file 1 [file ijerph-17-00503-s001.pdf]

International Journal of
Environmental Research and Public Health

MDPI

Supplementary information

Article

Photochemical Generation of Methyl Chloride from Humic Aicd: Impacts of Precursor Concentration, Solution pH, Solution Salinity and Ferric Ion

Hui Liu *, Yingying Pu, Tong Tong, Xiaomei Zhu, Bing Sun and Xiaoxing Zhang

College of Environmental Science and Engineering, Dalian Maritime University, Dalian 116026, China; py1120181596@dlmu.edu.cn (Y.P.); tongtong@dlmu.edu.cn (T.T.); zhuxm@dlmu.edu.cn (X.Z.); sunb88@dlmu.edu.cn (B.S.); zhangxiaoxing@dlmu.edu.cn (X.Z.)

* Correspondence: liuhui@dlmu.edu.cn

Received: 20 November 2019; Accepted: 09 January 2020; Published: date

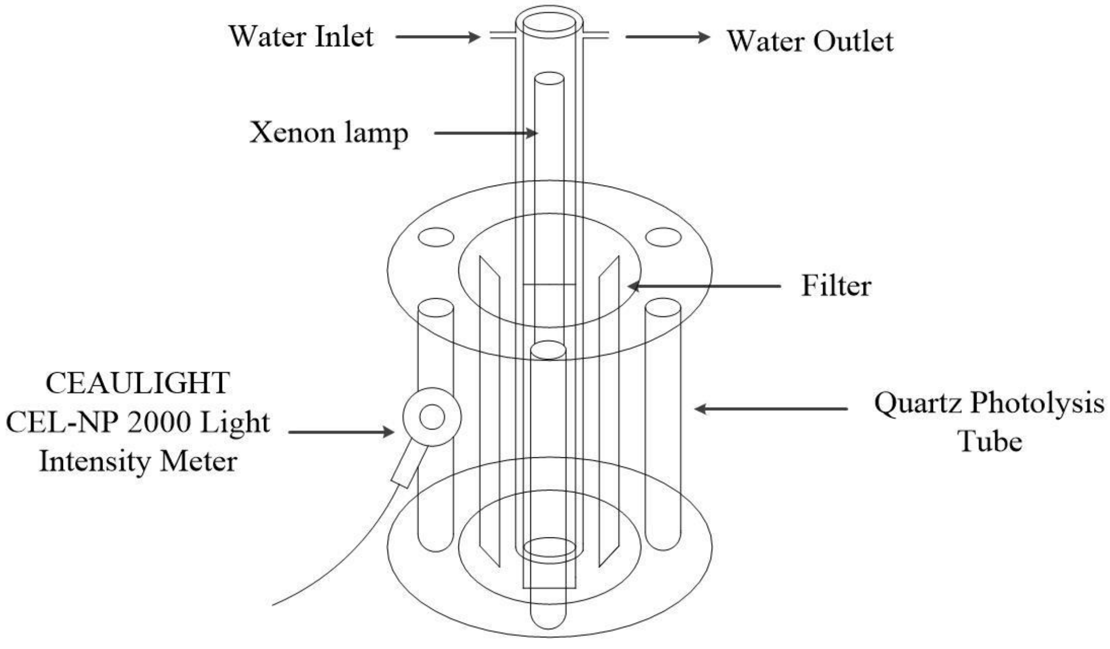

Figure S1. Schematic of the device used for irradiation. It is a so-called merry-go-round photo-reactor. Xenon lamp was placed in a quartz jacket where cold water (10 °C) was driven by a cooling circulating pump to take away the heat of the infrared radiation. Eight pieces of filters were placed around the jacket to cut off the light with the wavelength below 290 nm. A light intensity meter (CEAULIGHT CEL-NP 2000) was used to measure the light intensity at the position where to place the quartz tube.

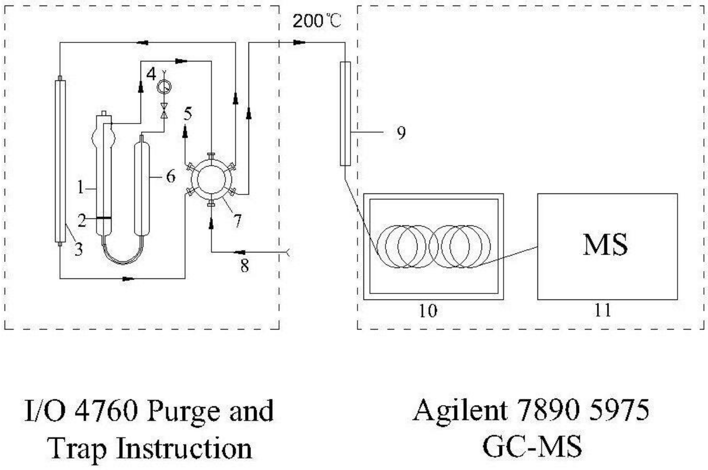

Figure S2. Illustration of the purge-and trap device and GC-MS. 1- sample tube; 2- glass sieve tube; 3- trap well; 4- purge gas inlet; 5- exhaust; 6- reservoir bottle; 7- valve; 8- GC carrier gas; 9- heating transmission line; 10- GC; 11-MS

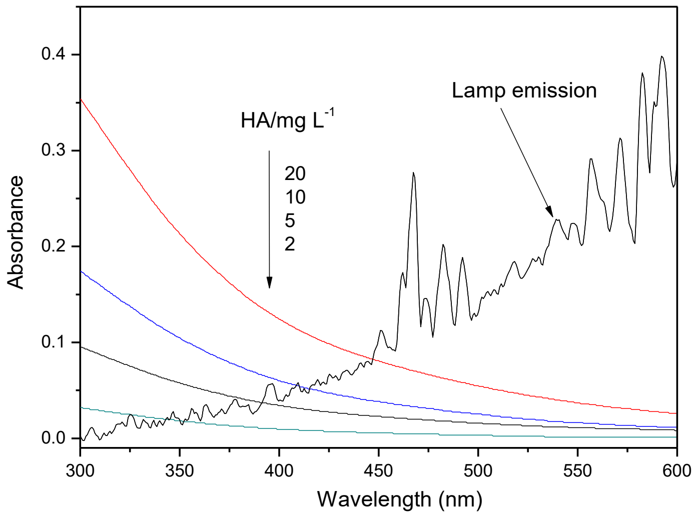

Figure S3. HA absorption and Xenon lamp emission spectrum.

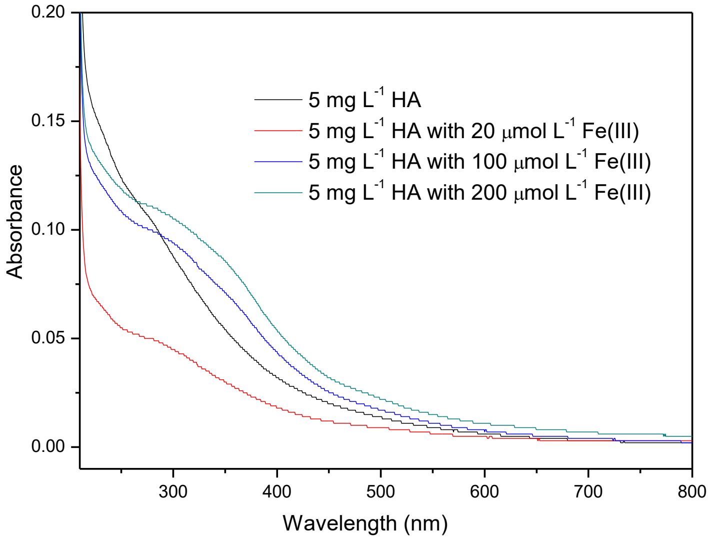

Figure S4. UV-vis spectra of HA in the presence of Fe(III). The absorbance at 254 nm often representing the aromatic structures in HA decreased after adding Fe(III), showing the loss of HA in the solution which was estimated to be 55% by adding 20 μmol L^{-1} Fe(III). With Fe(III) increasing, the absorbance increased as well, especially at the range of 300-350 nm which could be attributed the complex of Fe(III) species.

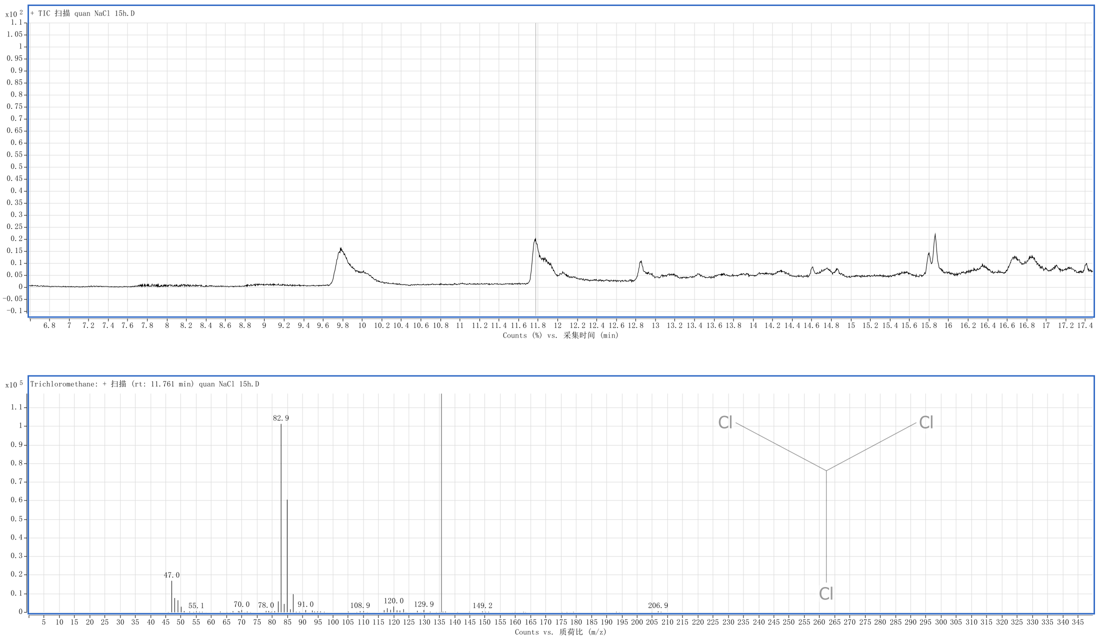

Figure S5. Chromatogram of the gases from the solutions containing 5 mg L^{-1} HA, 0.5 mol L^{-1} Cl^- and 600 μmol L^{-1} Fe(III) under irradiation of 15 h, and the MS spectra of trichloromethane.

© 2020 by the authors. Submitted for possible open access publication under the terms and conditions of the Creative Commons Attribution (CC BY) license (http://creativecommons.org/licenses/by/4.0/).
